# A Processing-in-Memory Architecture Programming Paradigm for Wireless Internet-of-Things Applications

**DOI:** 10.3390/s19010140

**Published:** 2019-01-03

**Authors:** Xu Yang, Yumin Hou, Hu He

**Affiliations:** 1 School of Computer Science and Technology, Beijing Institute of Technology, Beijing 100081, China; yangxu@tsinghua.edu.cn; 2 Institute of Microelectronics, Tsinghua University, Beijing 100084, China; hou-ym12@mails.tsinghua.edu.cn

**Keywords:** Processing-in-Memory, programming paradigm, Internet of Things

## Abstract

The widespread applications of the wireless Internet of Things (IoT) is one of the leading factors in the emerging of Big Data. Huge amounts of data need to be transferred and processed. The bandwidth and latency of data transfers have posed a new challenge for traditional computing systems. Under Big Data application scenarios, the movement of large scales of data would influence performance, power efficiency, and reliability, which are the three fundamental attributes of a computing system. Thus, changes in the computing paradigm are demanding. Processing-in- Memory (PIM), aiming at placing computation as close as possible to memory, has become of great interest to academia as well as industries. In this work, we propose a programming paradigm for PIM architecture that is suitable for wireless IoT applications. A data-transferring mechanism and middleware architecture are presented. We present our methods and experiences on simulation-platform design, as well as FPGA demo design, for PIM architecture. Typical applications in IoT, such as multimedia and MapReduce programs, are used as demonstration of our method’s validity and efficiency. The programs could successfully run on the simulation platform built based on Gem5 and on the FPGA demo. Results show that our method could largely reduce power consumption and execution time for those programs, which is very beneficial in IoT applications.

## 1. Introduction

We have entered the Era of Big Data, and the world is encountering the processing evolution of those Big Data. Existing systems used in Big Data processing are becoming less energy-efficient and fail to scale in terms of power consumption and area [1,2]. The widespread applications of the wireless Internet of Things (IoT) is one of the leading factors in the emerging of Big Data. Huge amounts of data need to be transferred and processed. Under Big Data application scenarios, the movement of large scales of data influences performance, power efficiency, and reliability, which are the three fundamental attributes of a computing system.

**Figure 1:**
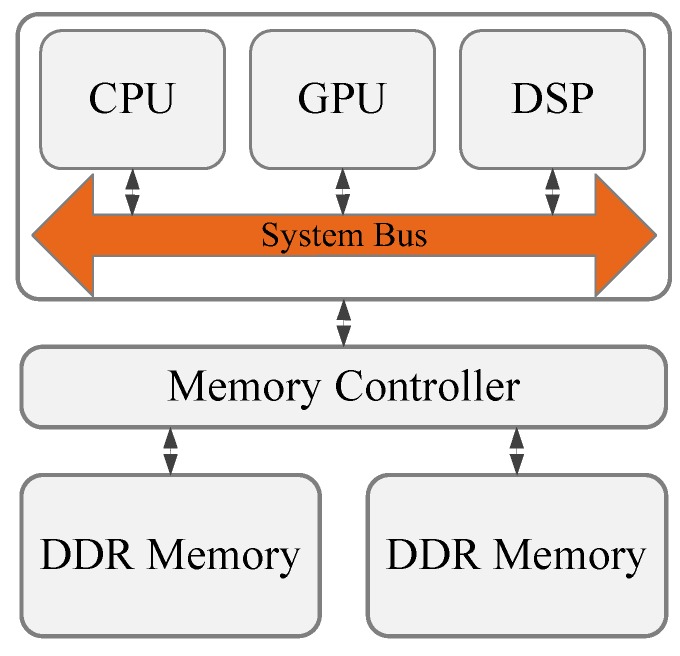
Traditional computing architecture.

The trend of the ever-growing number of applications of wireless IoT is leading to changes in the computing paradigm and, in particular, to the notion of moving computation to data in what we call the Processing-in-Memory (PIM) approach. A traditional computing architecture is shown in Figure 1. Computing units may include the CPU, GPU, and DSP. Data are transferred between the computing units and the main memory through the memory-hierarchy levels. The bottleneck of data processing for a traditional computing architecture is the bandwidth and latency of data transfer, since a large amount of data are stored in the DRAM [3,4]. Although processors have large caches and an embedded memory, there is an increasing number of data stored in DRAM for high-throughput applications (Big Data processing scenarios, as well as radar-signal processing, video/image data processing, deep learning, etc.). If we want to overcome the shortage of traditional computing architectures to better suit Big Data processing, we need to move some computation units to DRAM to exploit PIM technology. With the evolution of emerging DRAM technologies, PIM has now become of great interest to academia as well as different industries [5,6] after a period of dormancy. PIM prototypes always integrate simple processing units with DRAM arrays to minimize data movement and perform computation right at data’s home. This is in contrast to the movement of data toward a CPU independent of where it resides, as it is done traditionally. It is also proposed that data computation can be performed in caches, or persistent storage (Solid State Drive—SSD) [7].

**Figure 2:**
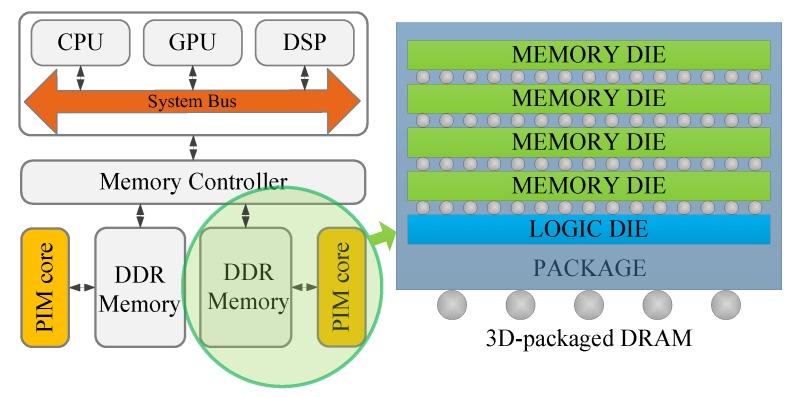
Processing-in-Memory (PIM) concept.

**Figure 3:**
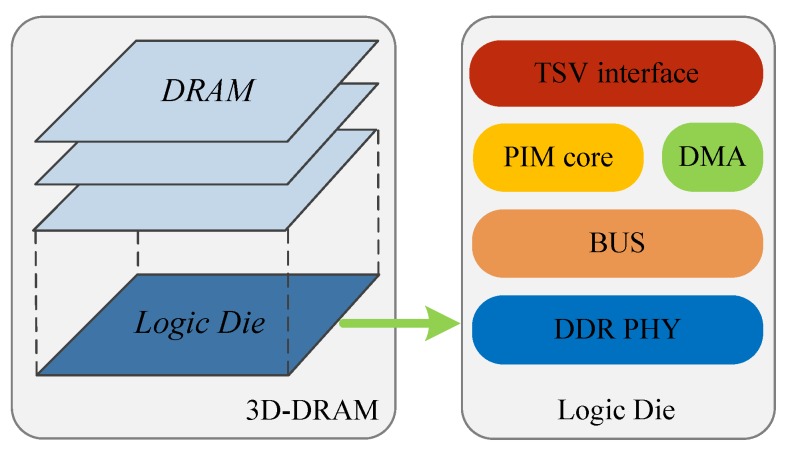
PIM device.

Figure 2 shows the PIM concept. On the basis of a traditional computing architecture, a computing unit (we call it the PIM core in this paper) is located near DDR memory. Three-dimensional packaging technology supports the integration of DDR memory and the PIM core (the circled parts in Figure 2). Three-dimensional packaging technology stacks heterogenous layers, including DRAM dice and a logic die, in a single chip, which is called a PIM device, as shown in Figure 3. The logic layer includes the PIM core, DMA, Through-Silicon Via (TSV) interface, BUS, and DDR PHY. Companies such as Micron and Samsung are dedicated to the exploration of 3D packaging technology. Products such as Hybrid Memory Cube (HMC) [8] and High Bandwidth Memory (HBM) [9,10] have already been released to markets. Three-dimensionally packaged memory provides a new approach to the memory system architecture. Heterogeneous layers are stacked with significantly more connections. TSVs enable thousands of connections of the stacked layers. The PIM core can quickly access the DDR memory. The HMC also provides a fast connection to the host CPU.

By adopting PIM, a PIM device becomes a small computing engine. With the PIM core fulfilling the majority of the computing tasks, the data-transfer rate between CPU and memory is largely reduced. In modern computing systems, load/store operation consumes much more power than data-processing operations. For the Intel XEON CPU, power consumed by the data transfer between CPU and memory is 19.5 times of that between CPU and the L1 cache [11]. Thus, PIM adoption largely reduces the power consumption of the computing system. By moving data-processing units to the memory, the burden of the CPU can be lightened, and the area of the CPU can also be reduced.

In typical IoT applications, huge amount of devices are connected to form a huge data transmissions and an interaction network. Data collected from end-devices might be raw, doubtful, and in large amounts. PIM could help in performing necessary end-device data preprocessing to provide a preliminary understanding of those collected data. Further compressing the amount of data needs to be transferred and exchanged, which might be very beneficial for IoT applications, where energy is very important.

In this work, we present our methods and experiences on simulation-platform design, as well as FPGA demo design, for a PIM architecture. The proposed programming paradigm was verified on both platforms. The following of this paper is organized as follows: Related works are discussed in Section 2. The target PIM architecture is introduced in Section 3. We provide details about our programming paradigm in Section 4. In Section 5, we describe our approach of the implementation of a simulator, and an FPGA demo, for the presented PIM architecture. The experiment results based on the simulation platform are given in Section 6. We show the application prospects of the PIM architecture in Section 7. Finally, we draw conclusions in Section 8.

## 2. Related Works

Many works have been done on PIM since the 1990s. EXECUBE [12], the first multinode PIM chip based on DRAM, was developed in 1992. During the same era, Intelligent RAM (IRAM) [13,14], Computational RAM (CRAM) [15], Parallel Processing RAM (PPRAM) [16], FlexRAM [5], DIVA [17], Smart Memories [18], and Intelligent Memory Manger [19] were developed. IRAM was designed for multimedia applications that include large amounts of computation. CRAM integrates many simple one-bit data-processing elements at the sense amplifiers of a standard RAM to realize the high-speed execution of applications with high parallelism. Most PIM projects of that era share the same characteristics. The PIM chip always includes several simple processing cores located near the DRAM arrays. They all realize high-speed parallel data processing.

Though promising results were witnessed at that time, no widespread commercial product emerged because producing such PIM chips was very expensive. After decades of dormancy, the interest to study PIM has been revived. With the emergence of 3D-staking technology, Big Data workloads and distributed programming paradigm, a new concept of Near Data Processing (NDP) was proposed.

Recent studies on NDP include References [1,20,21,22,23], of which References [1,22,23] all propose an ARM-like core as the PIM core integrated in 3D-DRAM. Our research most resembles Reference [1]. We both propose an ARM-like PIM core and we used gem5 [24] to realize the simulation of the PIM architecture. McPAT [25] was applied to analyze power consumption. The difference is that Reference [1] focused on PIM design-space exploration, simply simulating the PIM core based on the gem5 simulator. In our research, we simulated the PIM computing system, including both the host processor and the PIM core. Workloads were assigned between host processor and the PIM core, and communication between them was realized.

The contributions of this paper are listed as follows:

- We propose a programming paradigm for PIM architecture. Drivers and APIs were implemented. An elaborate programming example is provided.
- We simulated a complete PIM computing system, including the host CPU and PIM cores, based on the gem5 simulator. We implemented the proposed programming paradigm using system calls.
- We built a board-to-board FPGA demo for the PIM architecture. The proposed programming paradigm was verified in this demo.
- We provide a performance comparison between the PIM computing architecture and traditional architectures.
- We show the application prospects of the PIM architecture, where our programming paradigm could also be utilized.

## 3. Target Architecture

**Figure 4:**
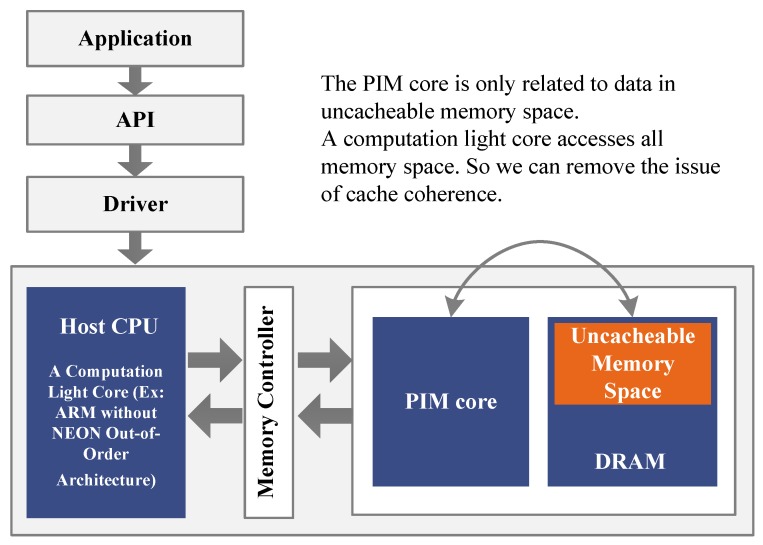
Design philosophy of the PIM architecture.

We first introduce the design philosophy of the PIM architecture. As shown in Figure 4, the PIM architecture (shown inside the large gray rectangle) should be composed of a host CPU, a PIM core, memory controller, and memory (DRAM in Figure 4). The host CPU should be a computation light processor. The host CPU, for example, can be an ARM without a NEON Out-of-Order core. According to the design methodology of PIM systems, the PIM core is integrated in the same chip with DRAM. The PIM core could be an SIMD machine, a GPU-like multithreading machine [4], a reconfigurable array, many core systems, etc. References [26,27] also states that an SIMD/VLIW/vector processor is fit for the data-processing unit in a PIM system. The host CPU and PIM cores share the same physical memory. The host CPU can access the whole memory space, making some memory space in the DRAM uncacheable. The PIM core uses the uncacheable memory space to run the program. The cached memory space is read-only for the PIM core. Users can control the PIM core through drivers. The software-development environment provides APIs for programmers.

**Figure 5:**
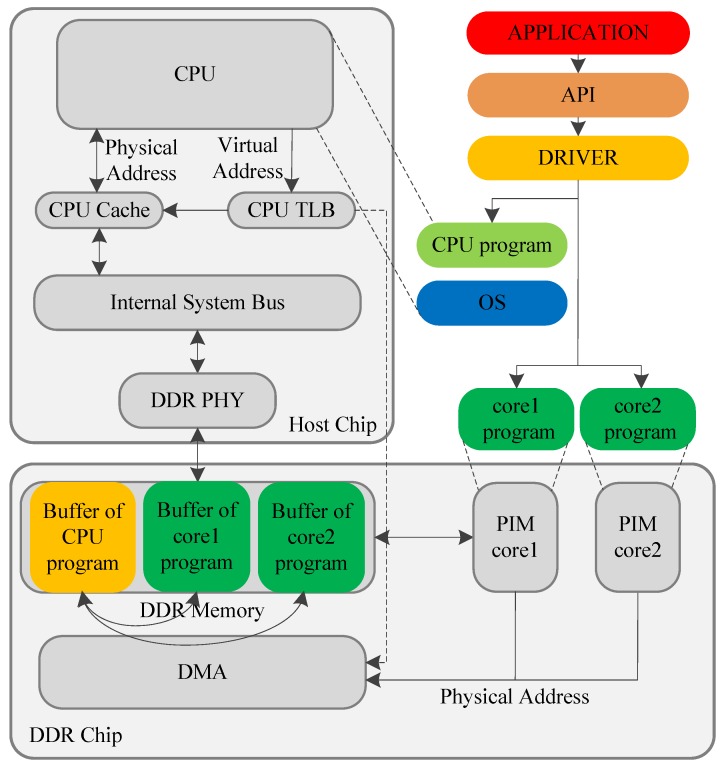
Presented PIM architecture.

Based on this design philosophy, we designed a PIM architecture as shown in Figure 5. The whole system consists of a host chip and a DDR chip. The host chip contains the host CPU, CPU cache, CPU TLB, internal system bus, and DDR interface. The DDR chip is a PIM device that contains the DDR memory, PIM core, and DMA. We used a simple ARM core as the PIM core, which could be configured to have one or two PIM cores. In the example presented in Figure 5, the number of PIM cores was set to two. More PIM cores can be placed in the logic layer of a 3D-packaged memory, but we limited the number to reduce simulation time. The host CPU is in charge of running the OS to control the whole system, while the PIM cores (core1 and core2) on the DDR chip focus on applications that need large amounts of computation and memory access. The CPU and the PIM cores can claim buffers in DDR memory for program execution, as shown in Figure 5. For the efficient transfer of large-scale data between the buffer of the CPU program and the buffers of PIM core program, a DMA was integrated in the DDR chip.

## 4. Programming Paradigm

In order to fully explore the potential of the PIM computing architecture, we have designed a programming paradigm, which is discussed in detail in this section.

### 4.1. Task-Dividing Mechanism

First, a program should be analyzed to identify the behavior of each part.

As discussed before, in the presented PIM computing architecture, there is one host CPU and one PIM device. The host CPU is mainly in charge of running OS, JVM, and control-intensive tasks. The PIM cores in the PIM device are used to deal with a large scale of data processing. So, according to the results of program analysis, a program is divided into two parts: control-intensive tasks and data-intensive tasks. Control-intensive tasks are assigned to the host CPU, while data-intensive tasks are assigned to PIM cores. The division of tasks should follow predefined rules to ensure the granularity of those tasks in an appropriate level, thus reducing unnecessary frequent intercommunications between the host CPU and PIM cores.

### 4.2. Data-Transferring Mechanism

After task division, the data flow of the program between host CPU and PIM cores is clear. Then, the data-transferring mechanism for this program should be designed.

**Figure 6:**
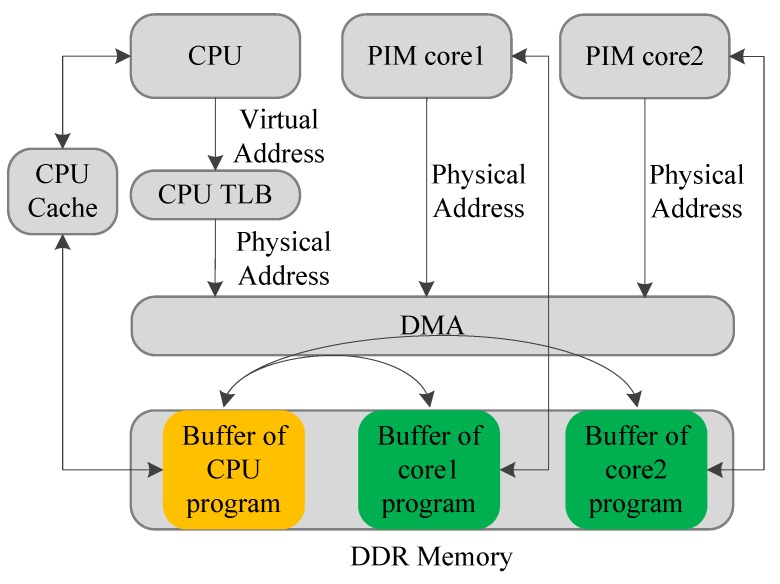
Data-transferring mechanism.

The data-exchange mechanism between the host CPU and the PIM cores is illustrated in Figure 6. In the target architecture, the CPU and the PIM cores share the same physical memory space. The CPU can access the memory by two means. Generally, data are transferred between CPU and memory through the CPU cache. The CPU can also transfer large-scale data to the PIM memory space through the DMA. The DMA does not adhere to the memory-consistent protocol, so the CPU has to perform a special process before exchanging data with the PIM cores. Before the CPU transfers data to the PIM cores, it should flush the cache data into the memory. After the PIM cores transfer data to the CPU, the CPU has to invalidate the corresponding cache line, and refresh the data from the memory. The PIM cores can directly access the DDR memory.

**Figure 7:**
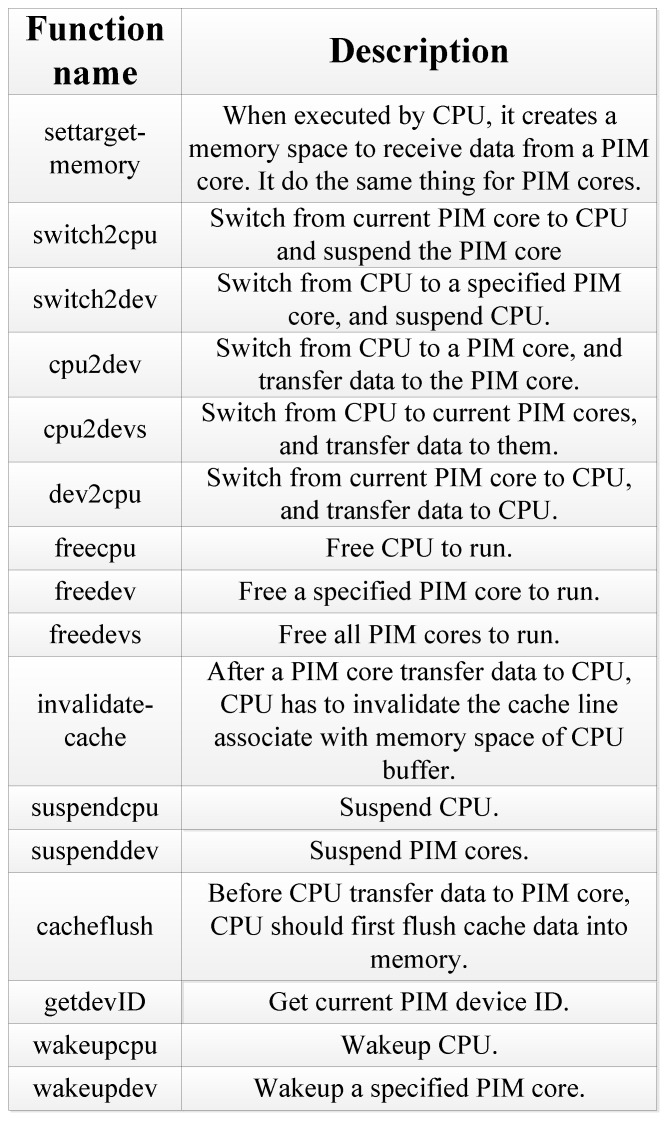
Basic functions supporting the proposed programming paradigm.

Some basic functions are designed to realize the operations stated above, as shown in Figure 7. With the help of those functions, the data-transferring mechanism between the host CPU and PIM cores is designed and decided.

### 4.3. Software-Level Architecture

**Figure 8:**
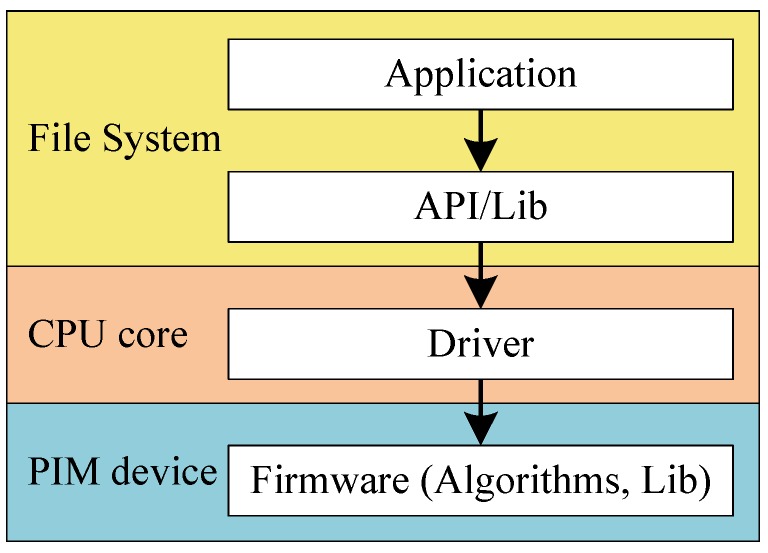
Software-level architecture of the PIM computing system.

The software-level architecture of the PIM computing system is shown in Figure 8. This includes application, API, driver, and firmware. User application programs and API are all located in the file system. API codes are substantial library functions. Drivers run on the host CPU. Through the drivers, the CPU can interact with the firmware running on the PIM device, and control the operation the PIM device.

Firmware is a microsystem running on the PIM device. Note that the PIM device does not run an operating system. The firmware allows the PIM device to interact with the user. It makes specific allocation of the whole address space. The firmware includes application-targeted algorithms. It provides plenty of functions that can be called in user applications. Users can call these functions by calling the PIM_context, which we define below. PIM_context includes three pointers. User_func_pointer points to the user function. PIM_func_pointer points to PIM firmware functions. Vars_pointer points to global variables used in these functions. PIM_context enables the PIM device to receive CPU-transferred programs and data. This helps improve users’ programming efficiency. In the firmware operation process, firmware is started at first. Then, it waits until the data and algorithm are configured. Firmware uses the data to fulfil the algorithm execution. After execution is finished, it informs the driver of the end of execution, and stops working.

typedef struct{

        void *user_func_pointer;

        void *PIM_func_pointer;

        void *vars_pointer;

}PIM_context_t;

PIM drivers provide the following functions:

- **Firmware download:** The PIM device receives firmware sent by the user and downloads it to a specified location. Then, it frees the PIM device to run the firmware.
- **Data transfer:** This includes data send and receive. After firmware download is finished, data sent by a user are transferred to the PIM device and stored in a specific firmware location. After computation is finished, the specific length of the data is obtained from the specific location of the firmware, and then, the data are sent to the user.
- **Algorithm configuration and execution:** When the firmware is downloaded, the user can decide which algorithm the PIM device will run, and instruct the PIM device to start execution. The algorithm can be provided by the firmware or by the users.
- **Status check:** The user can check the status of the PIM device during execution. Only the PIM device itself can update its status. PIM device status includes PIM_start, PIM_wait_data, PIM_check_alg, PIM_running, and PIM_finish.

APIs can be divided into user-mode APIs and kernel-mode APIs. Kernel-mode APIs provide an interface to call drivers. User-mode APIs encapsulate kernel-mode APIs, and are more convenient for users to use user-mode APIs. User-mode APIs provide the following functions (A means address, indicating pointer type):

- **File operation :** to obtain file size, and read file to buffer.
  get_file_size(A file)
  read_file(A buffer, A file)
- **Function transfer :** CPU transfer user functions to PIM device. This realizes input and output buffer management for the PIM device. Since the CPU may transfer multiple functions to the PIM device, we should specify the main function running on the PIM device by the entry pointer.
  build_buf(A Obuf, A entry, A Ibuf, len)
  free_buf(A buffer)
- **Driver interaction :** The CPU obtains the PIM device information, and updates the firmware on the PIM device.
  find_device(A PIM_device)
  update_firmware(PIM_device, A buffer, len)
- **Operational configuration :** The CPU configures the PIM device to conduct computation. It chooses the algorithm on the PIM device firmware, sends and collects computation data, obtains the computation status of the PIM device, and waits for the PIM device computation to finish.
  set_algorithm(PIM_device, alg)
  get_data(PIM_device, A buffer, len)
  put_data(PIM_device, A recv_buffer, len)
  check_status(PIM_device)
  wait(PIM_device)

### 4.4. Programming Instructions

**Figure 9:**
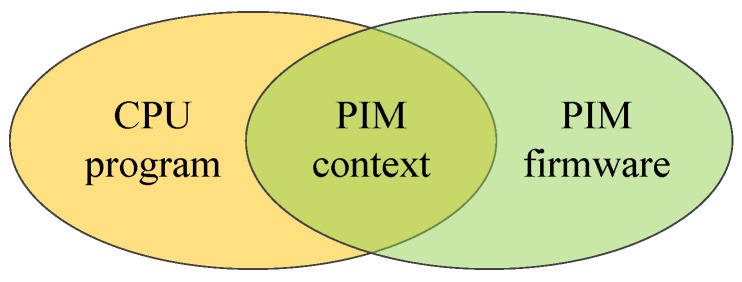
Program structure for the PIM computing architecture.

In a program running on PIM computing architecture, the host CPU and PIM device interact with each other through PIM_context, as shown in Figure 9. When programming, users should use the PIM_func prefix to indicate the function running on PIM device, and use the PIM_vars prefix to indicate global variables used by PIM device functions. The PIM entry function and PIM variables are included in PIM_context. Through PIM_context, PIM device can call user functions, as well as firmware functions. Users should follow predefined rules to call functions and to use global variables.

Execution of a program targeting the PIM computing architecture includes the following steps: find PIM device → send firmware function → set algorithm → send data → wait for computation finish → receive data. A program example is given below.

/*A program example*/

/*some parameters are omitted,

data types are omitted*/

 

*//callee on PIM device*

PIM_func int callee()

 

*//entry function run on PIM device*

PIM_func int entry(PIM_context)

{

 PIM_CALL_USER_FUNC(PIM_context, callee)

}

 

*//firmware file*

#define FIRMWARE “PIM_EXEC.bin”

 

int intput[n];

int output[n];

int main()

{

 *//get PIM device*

 find_device(&PIM_dev);

 

 *//send program run on PIM device*

 size = get_file_size(FIRMWARE);

 buffer = malloc(size);

 read_file(buffer, FIRMWARE);

 update_firmware(PIM_dev, buffer, size);

 free(buffer);

 

 *//set algorithm*

 set_algorithm(PIM_dev, ALG);

 

 *//send function and variables*

 buffer = 0;

 size = build_buf(&buffer, entry, input)

 get_data(PIM_dev, buffer, size);

 free_buf(buffer);

 

 *//wait for computation finish*

 wait(PIM_dev);

 

 *//collect computation data*

 put_data(PIM_dev, output);

}

 

## 5. Evaluation Platform Design

### 5.1. Simulator Based on Gem5

We have built a simulation platform based on gem5 [24] to evaluate the PIM computing architecture, and to verify the proposed programming paradigm. During the implementation of this platform, we experienced new challenges. Gem5 [28,29] is an open-source platform for computer-system architecture research, encompassing system-level architecture as well as processor microarchitecture. Gem5 is written in the C++ and Python languages. It has several interchangeable CPU models, and can support the simulating of multiprocessor systems. Gem5 has event-driven memory systems. However, the Gem5 simulator does not support the EPIC architecture processor models and VLIW ISA simulation.

A fast simulation methodology is crucial for exploring a sufficiently broad spectrum of applications and relevant design points. An evaluation method proposed in Reference [4] is first gathering hardware performance and power statics during execution on the current hardware. Then, the data are fed into a machine-learning model that predicts the performance and power on future PIM and host hardware configurations. However, this method is not accurate.

Recently, AMD proposed a work to explore the PIM design space [1]. They used gem5 to simulate the PIM architecture. They used a minor CPU model and a gem5 DRAM module to run MapReduce applications. In their simulation framework, the host processor was not included because gem5 does not yet support such systems.

Gem5 can now support multicore and multisystem simulations. In conjunction with full-system modeling, this feature allows the simulation of entire client–server networks. For multicore simulations, the cores are constrained to use the same CPU model. In the proposed PIM architecture, we put the PIM core near the memory, while the CPU still accessed the memory through memory hierarchy. The CPU and PIM core access memory differently. In gem5, AtomicSimpleCPU is the only CPU model that supports the fast memory (fastmem) access method, which most resembles the memory access method of the PIM core. AtomicSimpleCPU is the simplest CPU model in gem5. It finishes one instruction in one cycle. AtomicSimpleCPU can be used as the PIM core. Different CPU models should be used to simulate the host CPU. We have to modify the gem5 simulator to realize this architecture.

**Figure 10:**
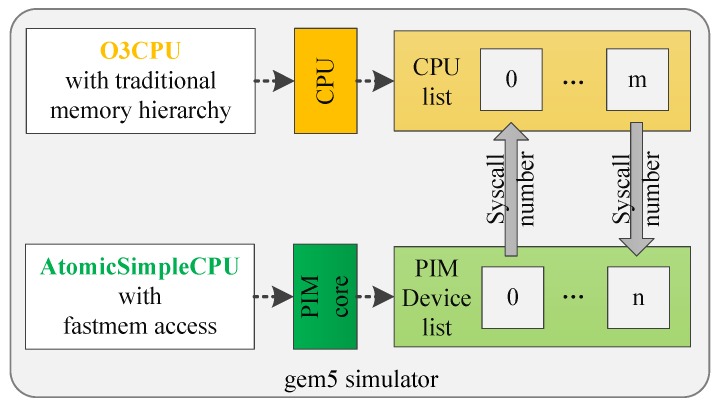
Gem5 simulator of the PIM computing architecture.

Figure 10 shows the gem5 simulation model we designed. The host CPU was implemented based on the O3CPU model. O3CPU is an Out-of-Order CPU model with five pipeline stages. We used the AtomicSimpleCPU as the PIM core. The number of CPUs and PIM cores can be set to an arbitrary value. The basic functions shown in Figure 7 are realized in the form of system calls on gem5 to support the programming paradigm. CPUs and PIM cores can communicate with each other using system calls.

### 5.2. Board-to-Board FPGA Demo

We also built a board-to-board FPGA demo to verify the proposed programming paradigm. We used two Xilinx ZC706 evaluation boards to build the proposed PIM architecture. One of the boards worked as the master board, and the other board worked as the slave board. The Xilinx ZC706 board embraces a ZYNQ device, which integrates an ARM Cortex-A9 processor with FPGA in a single chip. We used the ARM Cortex-A9 on the master board that works as the host CPU. A self-designed ARM-compatible processor, working as the PIM core, was implemented on the FPGA of the slave board.

**Figure 11:**
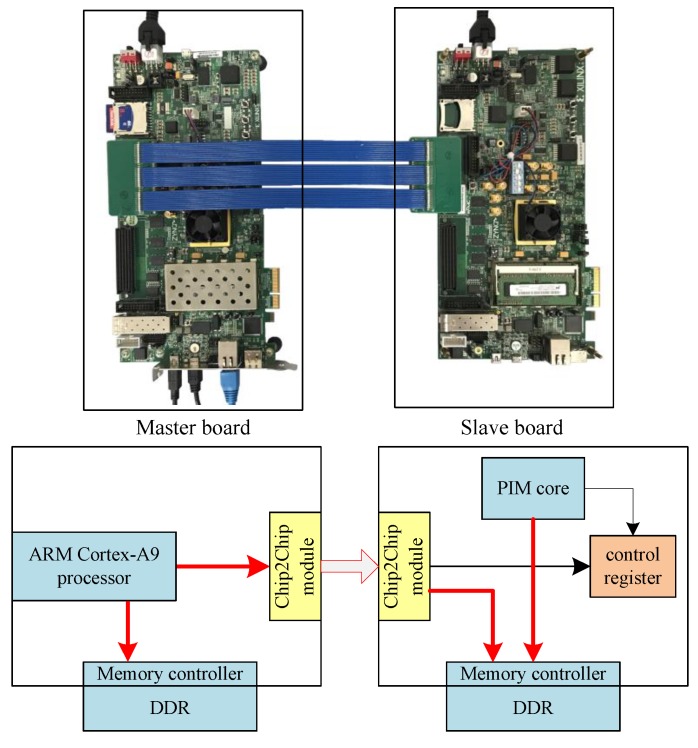
FPGA demo.

As shown in Figure 11, the two boards can be connected by an FPGA Mezzanine Card (FMC) Interface. Host board and slave board can communicate with each other through the chip2chip module, which is software IP supported by the ZC706 board. In the PIM computing system, the slave board can be regarded as a device of the master board. The master board can access the DDR and the control register of the slave board through the chip2chip module by accessing mapped address space. The host CPU can send control signals through the chip2chip master module to the chip2chip slave module. The PIM core receives the control signals and starts working. During execution, the PIM core can access the DDR though the AXI bus. When execution is finished, the slave board can send interrupt signals to the AXI interrupt controller, and then to the host CPU. The host CPU collects the result data from DDR.

On this FPGA demo, we were able to verify that the proposed programming paradigm is feasible and efficient.

## 6. Experiments

### 6.1. Experimental Framework

Several experiments were conducted on the gem5 simulation platform. We used the Mpeg2decode and MapReduce programs to test our programming paradigm.

Mpeg2decode programs convert Mpeg2 video bit streams into uncompressed video, so there is much data-intensive work. We chose two code stream files for Mpeg2decode programs to process. The two files were centaur_1.mpg and cinedemo.m2v. Centaur_1.mpg was a black and white image, and cinedemo.m2v was a color image. The color image had a larger bitmap size, and was more time-consuming to decode. These two files are typical and illustrative enough as test files for Mpeg2decode programs.

MapReduce is a popular programming framework for parallel computing of large-scale datasets. We realized MapReduce algorithms in C language. Four testbenches, wordcount, histogram, stringmatch, and matrix-multiply, were implemented. The four MapReduce testbenches were ported to the PIM computing architecture based on the proposed programming paradigm. Note that when the dataset size was 10 MB, the matrix-multiply program was estimated to run days to finish computing on this evaluation platform. So, it was assigned to compute just 10 rows of the matrix under all datasets.

To provide a comprehensive performance comparison between the PIM architecture and other traditional architectures, four architecture models, including CPU-only, PIM, PIM2, and GPU, are referred to in the experiments. Configuration details of the four models are shown in Table 1. CPU-only is a traditional CPU architecture that is modeled by the O3CPU model in gem5. For the PIM model, the host CPU is configured the same as CPU-only model, and the PIM core is configured as the PIM core shown in the table. PIM core is modeled by AtomicSimpleCPU in gem5. For the PIM2 model, the host CPU is also configured in the same way as the CPU-only model, and it had two PIM cores. These three models were all implemented on gem5 simulator. The target ISA is ARM ISA, and the compiler we used was gcc linaro-4.7-2013.06-1. We used McPAT [25] for power analysis. McPAT is fed with the statistic generated by gem5 simulator to provide power-analysis results. Since GPU is widely used in the Big Data domain, we also provide performance comparison with GPU model. The GPU model we used is NVIDIA GeForce GTX480, based on the GPGPU-sim simulation platform. The GPUWattch model in GPGPU-sim is used for power evaluation. GPUWattch is a modified version of McPAT, dedicated for GPU architecture.

### 6.2. Results

#### 6.2.1. Mpeg2decode Programs—CPU-Only vs. PIM

The first test case is Mpeg2decode. We first evaluated the performance of CPU-only and PIM architecture with different CPU cache sizes. Only one level CPU cache was set in this experiment. Two code stream files, centau_1.mpg and cinedemo.m2v, were tested on the CPU-only model and PIM model.

**Figure 12:**
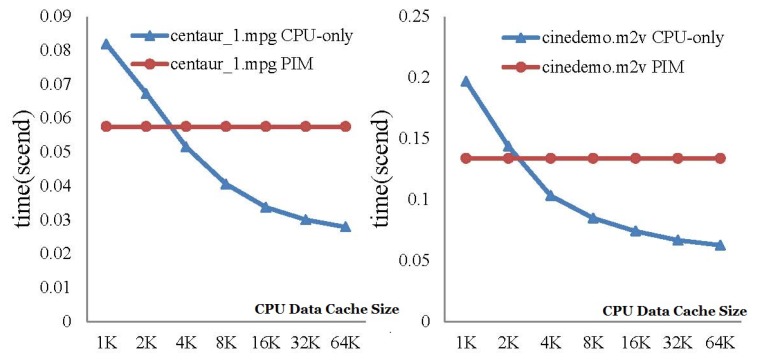
Execution-time comparison between the CPU-only and PIM models running Mpeg2decode programs, with an increasing CPU data cache size.

Figure 12 shows the execution-time comparison between CPU-only and PIM model with an increasing CPU data cache size. For the CPU-only model, performance improved with the increase of the CPU data cache size, while the performance of the PIM architecture was not affected by the size of the CPU data cache. The results show that the PIM architecture did not require a large cache size to achieve high performance. For the CPU-only model, a larger data cache brought better performance, since more data could be locally processed. This result also demonstrates the significance of processing data at the data’s home. When the data cache size was 64 kB, the performance of the CPU-only model was about twice of the PIM architecture. This is because the speed of the O3CPU was twice that of the AtomicSimpleCPU in gem5.

**Figure 13:**
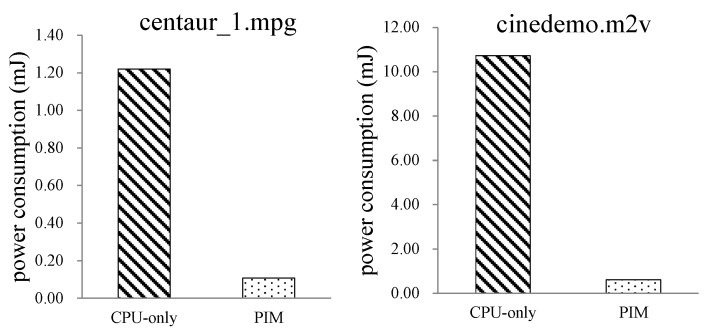
Comparison of processor power consumption between the CPU-only and PIM models running Mpeg2decode programs.

Processor power consumption, which includes the power consumption of the processor core and L1 cache, is shown in Figure 13. The results show that the power consumption of the PIM model was much less than that of CPU-only model. On average, the PIM architecture reduced processor power consumption by 93% compared to the CPU-only model.

Comparison of cache power consumption is shown in Table 2. Comparison of bus power consumption is shown in Table 3. It is shown that the cache power consumption of the PIM model was much smaller than that of the CPU-only model. On average, cache power consumption of the CPU-only model was about $10^{6}$ times of that of the PIM architecture. Bus power consumption of the CPU-only model was about $10^{5}$ times of that of the PIM model.

#### 6.2.2. MapReduce Programs—CPU-Only vs. PIM

The second test case was the MapReduce algorithm. The dataset processed by the four MapReduce programs was about 10 MB for all.

**Figure 14:**
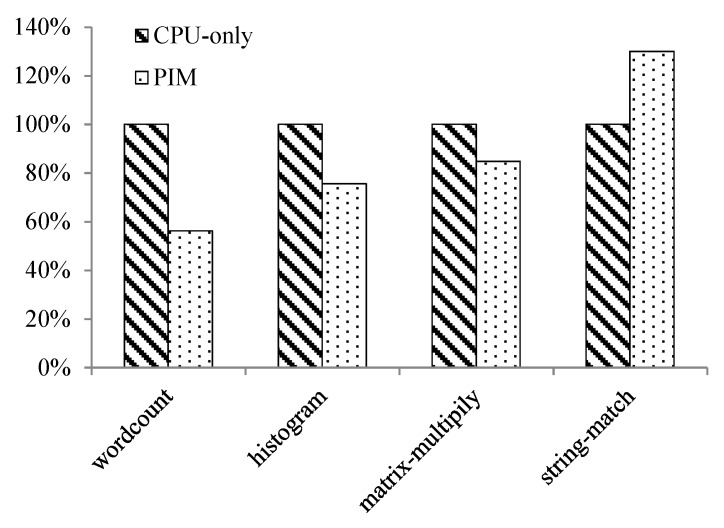
Execution-time comparison between the CPU-only model and PIM model running MapReduce programs.

Figure 14 shows the execution-time comparison between the CPU-only and PIM models running the four MapReduce programs. We assumed the time used by the CPU-only model was 100%. We can see from Figure 14, that for wordcount, histogram, and matrix-multiply, the PIM architecture reduced runtime by 44%, 24%, and 15%, respectively. For string-match, PIM architecture ran 30% longer than the CPU-only model. The reason is the disadvantage of the CPU-only model being long memory access delay. For the programs that require frequent memory access, the PIM architecture outperformed the CPU-only model, while for those programs that do not access memory that often, PIM might be slower than the CPU-only model. This result can also be partly attributed to the performance disparity of the AtomicSimpleCPU and O3CPU models in gem5. In other words, in the PIM model, the PIM core ran slower than the host CPU. Table 4 shows the memory access latency of the four programs in the CPU-only model. As we can see, memory access latency takes large percentage of the total runtime for wordcount, histogram, and matrix-multiply, while for string-match, memory access latency was not as significant as the other three programs. Thus, the PIM model showed performance loss when running string-match.

**Figure 15:**
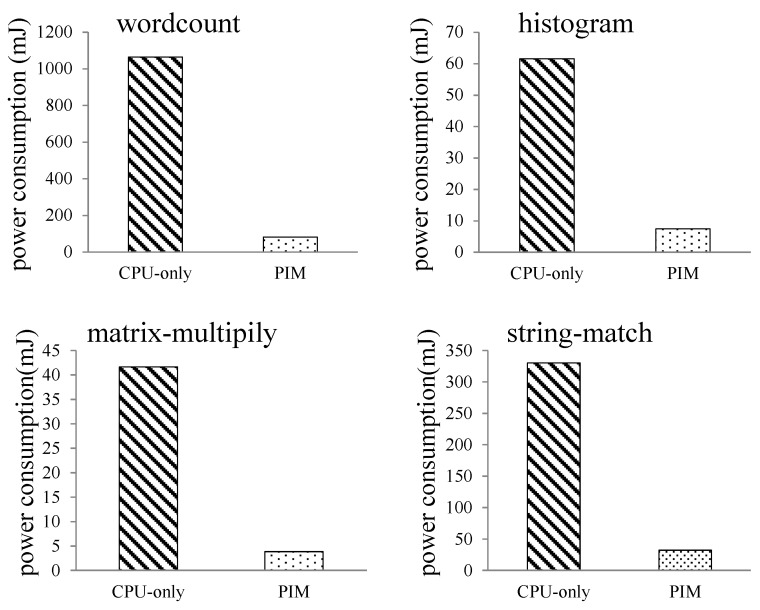
Comparison of processor power consumption between the CPU-only model and PIM model running MapReduce programs.

Comparison of processor, cache, and bus power consumption is shown in Figure 15, Table 5 and Table 6 separately.

As shown in Figure 15, for the four programs, processor power consumption of the PIM model was reduced by 92.4%, 88.6%, 90.7% and 90.3%, respectively, than the CPU-only model. Table 5 shows that, for the four programs, cache power consumption of CPU-only is $10^{3}$–$10^{4}$ times of that of PIM model. We can see from Table 6 that bus power consumption of the CPU-only model was about $10^{4}$ times that of the PIM architecture. PIM architecture reduced processor, cache, and bus power consumption to a large extent compared to the CPU-only model.

The experimental results demonstrate the characteristics of the PIM architecture. Since the PIM core directly accesses memory, it reduces the total cache and bus access. So, cache and bus power consumption is reduced. This result shows that the PIM architecture is suitable for applications processing large datasets and requiring frequent memory access.

#### 6.2.3. MapReduce Programs—CPU-Only vs. PIM vs. PIM2

In this experiment, we fed different dataset sizes to the MapReduce programs. When input data size increased from 1 to 10 MB, the performance and power consumption of the CPU-only, PIM, and PIM2 models was evaluated.

**Figure 16:**
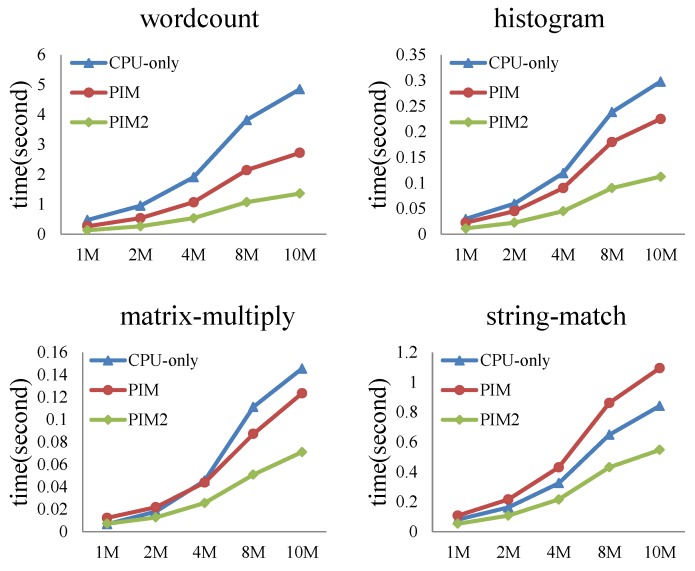
Execution-time comparison between the CPU-only, PIM, and PIM2 models running MapReduce programs with increasing datasets.

Figure 16 shows the execution-time comparison of the three models. For wordcount and histogram, the run time of the CPU-only model was always longer than the PIM and PIM2 models. The PIM2 model showed better performance than the PIM model. With the increase of the dataset size, the gap between the three models becomes larger, and the advantage of the PIM architecture becomes more evident. For matrix-multiply, the run time of the three models increased when the data size expanded. The run time of the PIM model became shorter than the CPU-only model when the dataset was larger than 4 MB. The PIM2 model always ran faster than the CPU-only model and PIM model. For string-match, the run time of the PIM model was always longer than the CPU-only model. The reason is that memory access is less frequent in this program, as shown in Table 4, while the PIM2 model showed better performance than the CPU-only model.

**Figure 17:**
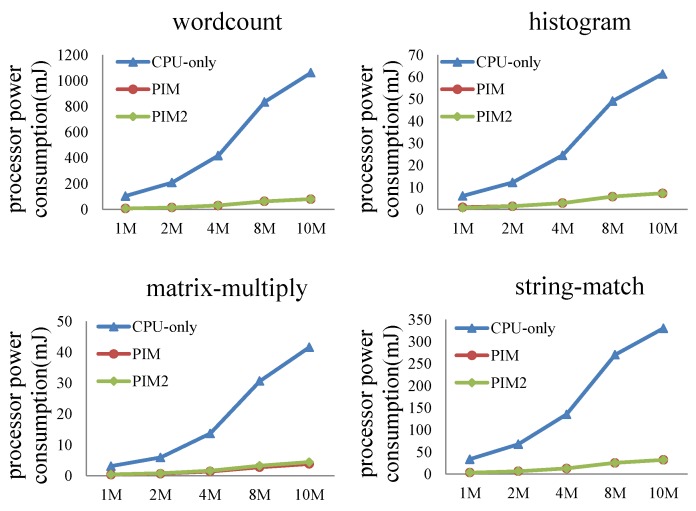
Comparison of processor power consumption between the CPU-only, PIM, and PIM2 models running MapReduce programs with increasing datasets.

Comparison of processor power consumption is shown in Figure 17. For the four programs, processor power consumption of the PIM model and PIM2 model were almost the same, since two identical cores share the work that was previously done by a single core. The energy consumed should be approximate. Processor power consumption of the PIM model and PIM2 model slightly increased when the data size expanded. Processor power consumption of the CPU-only model running the four programs increased substantially. This result shows that PIM architecture can largely reduce processor power consumption.

**Figure 18:**
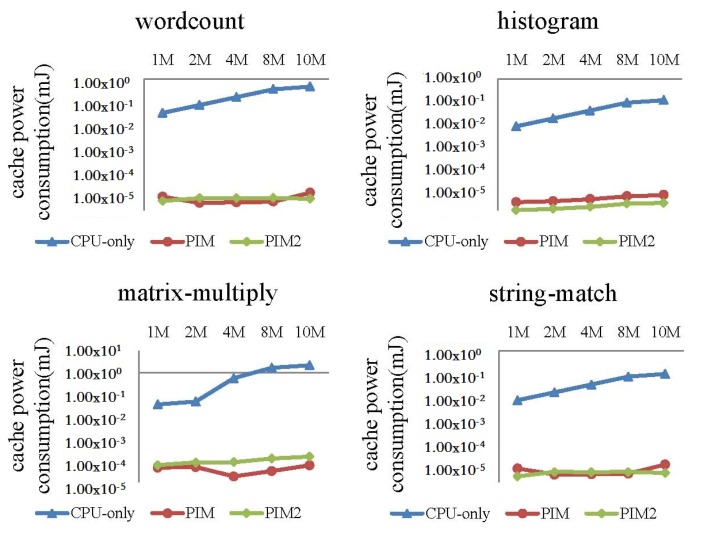
Comparison of cache power consumption between the CPU-only, PIM, and PIM2 models running MapReduce programs with increasing datasets.

**Figure 19:**
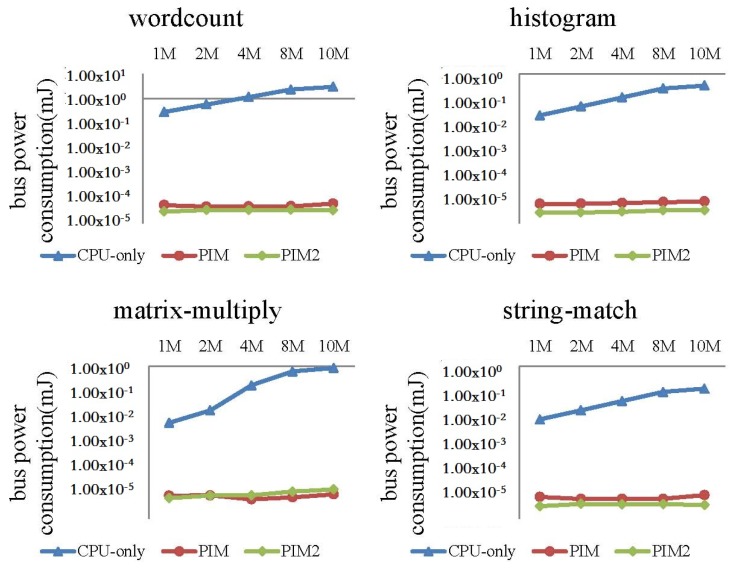
Comparison of bus power consumption between CPU-only, PIM, and PIM2 models running MapReduce programs with increasing datasets.

Comparisons of cache and bus power consumption are shown in Figure 18 and Figure 19, respectively. Logarithmic co-ordinates are adopted in these two figures. Cache and bus power consumption of the CPU-only model increased to a large extent for the four programs, while cache and bus power consumption of the PIM model and PIM2 model were much smaller comparatively.

**Figure 20:**
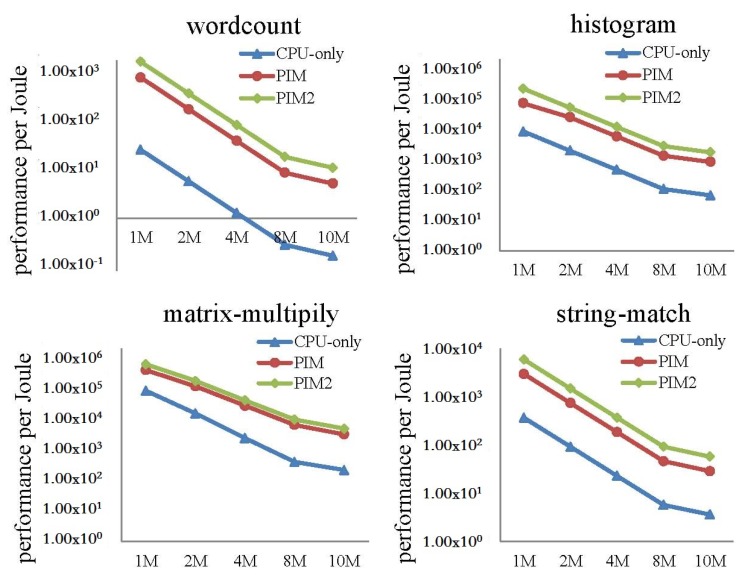
Performance per Joule comparison between the CPU-only, PIM, and PIM2 models running MapReduce programs with increasing datasets.

Figure 20 shows the performance per Joule of the three models. Logarithmic co-ordinates were adopted in this figure. The result is the ratio of reciprocal value of total runtime and average energy. We can see from the figure that performance per Joule of the three models decreased with increasing input data size. However, the PIM and PIM2 models showed about one order of magnitude higher performance per Joule than the CPU-only model. The PIM2 model showed better performance per Joule than PIM model.

From the experimental results, we can conclude that the run time of the PIM architecture is largely affected by the nature of the application and input dataset size. For the programs requiring frequent memory access, the PIM architecture can improve program performance. For all the tested applications, PIM architecture reduces processor, cache, and bus power consumption to a large extent. When the input dataset size increased, the advantage of the PIM architecture became more noticeable. Performance per Joule of the PIM and PIM2 models was also much higher than the CPU-only model. The PIM2 model could further improve the performance of the PIM architecture, and also showed better performance per Joule.

#### 6.2.4. MapReduce Programs —CPU-Only vs. PIM2 vs. GPU

In this experiment, we ran the four MapReduce programs on the CPU-only, PIM2, and GPU models, with the data size increasing from 1 to 10 MB. Run time and performance per Joule were evaluated.

**Figure 21:**
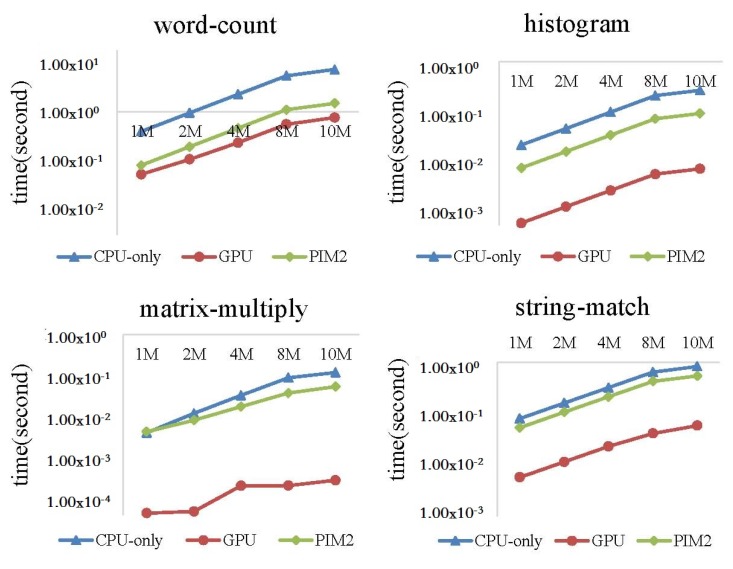
Execution-time comparison between the CPU-only, PIM2, and GPU models running MapReduce programs with increasing datasets.

Figure 21 shows the execution time of the four MapReduce programs on CPU-only model, PIM2 model and GPU model. We can see from the result that the GPU run much faster than CPU-only model and PIM2 model. For the wordcount program, the advantage of GPU was less evident than the other three programs. GPU showed the best performance running the matrix-multiply program. Matrix-multiply is quite computing-intensive, and there are many approaches to accelerate the algorithm on GPU. We adopted one of the approaches in our experiment, while the wordcount program was less suitable to run on GPU. For applications running on GPU, the assignment was divided into many small parts, with each part running on a thread of the GPU. However, for wordcount, we had to assign enough workload to each thread to simplify the final data-collecting work to the CPU.

**Figure 22:**
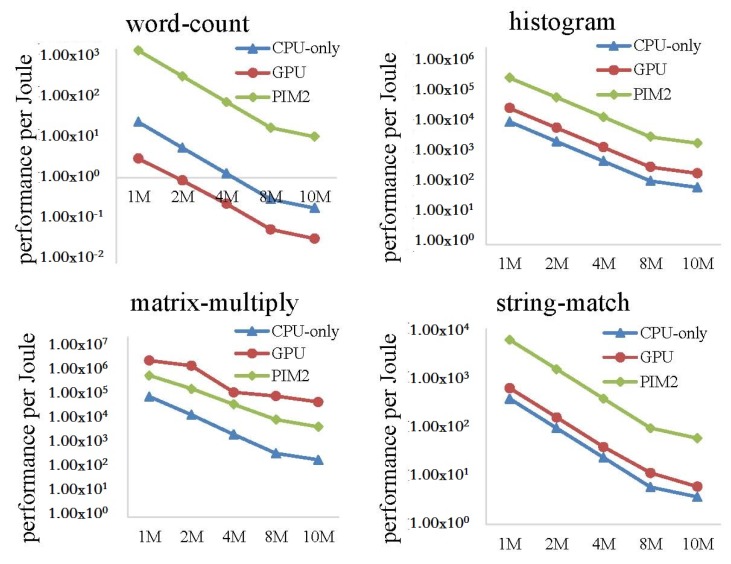
Performance per Joule comparison between CPU-only, PIM2, and GPU models running MapReduce programs with increasing datasets.

Figure 22 shows the performance per Joule of the three models. For matrix-multiply, GPU showed the best performance per Joule among the three models. Since this program quite suitable to run on GPU, and the performance advantage is evident enough to hide the high power of GPU. For wordcount, GPU showed the worst performance per Joule. Since the run time of GPU was close to the PIM2 model, as shown in Figure 21, and the power of the GPU was much higher than the CPU-only model and PIM2 model. For histogram and string-match, performance per Joule of the GPU was between the CPU-only model and PIM2 model. This result shows that the PIM architecture has an advantage over GPU for the applications that are comparatively less computing-intensive. The reason is that the PIM core is a general-purpose processor. By analyzing all the experimental results above, it can be predicted that the PIM architecture can achieve better performance for more computing-intensive tasks if the PIM core is replaced with specialized computing units.

## 7. Application Prospect

**Figure 23:**
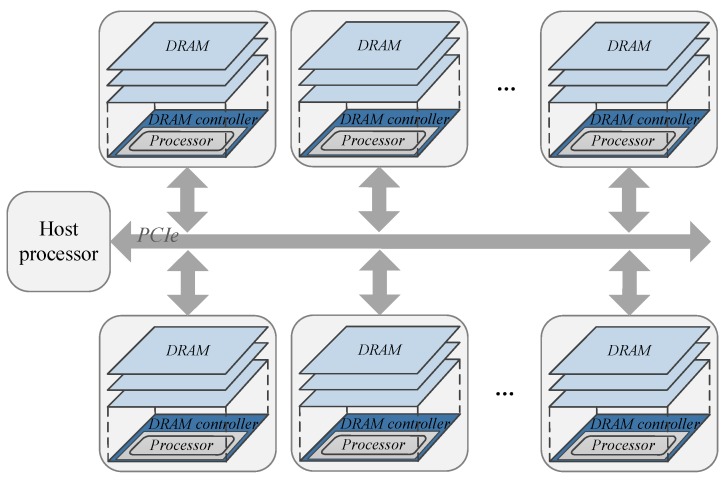
Server demo based on PIM computing architecture.

In this paper, we focused on studying a PIM computing architecture with one host CPU and one PIM device. A general-purpose ARM processor was used as the PIM core integrated in the PIM device. This architecture can be extended by allowing the host CPU access many PIM devices, as shown in Figure 23. The host CPU and the PIM devices can be connected via PCIe and SerDes. This computing architecture can be adopted by future servers to provide instant response service. The ARM processor can be replaced by other computing units according to the target application domain. The proposed programming paradigm can be also applied to the PIM computing architecture with multiple PIM devices. If a processor with embedded flash or ROM is used as the PIM core, firmware can be stored in the embedded flash/ROM to eliminate the operation of sending firmware to PIM devices every time. Users only need to update firmware when necessary. The PIM core can even be designed to run a simple operating system.

We believe that this programming paradigm might be very helpful for wireless IoT applications. IoT applications now encompass a lot of different domains, such as medicine, surveillance, transportation, and environmental protection. In those IoT applications, a lot of data might be collected in the end-devices. However, not all data, or not all raw data, need to be transferred or exchanged. Thus, PIM could be very beneficial in improving data-transmission and energy efficiency for those end-devices.

## 8. Conclusions

IoT applications are very popular today. It brings alive the Big Data scenario. Under such a scenario, huge amounts of data would be collected, transferred, and exchanged. In this paper, we proposed a programming paradigm for a PIM architecture suitable for wireless IoT applications. This programming paradigm with PIM could help perform data processing in the end-device near where the raw data are collected. Thus, preliminary understanding of the data could be given, and the amount of data needing to be transferred could be largely reduced and, hence, the required energy for IoT devices in data transmission. We have implemented several typical programs that are popular in wireless IoT applications based on the proposed programming paradigm. We ran these programs on a simulation platform, and on an FPGA demo. The proposed programming paradigm was proven to be feasible and efficient. The evaluation results based on the simulation platform were collected. The results show that, by adopting the proposed programming paradigm, we could exploit the benefits coming with a PIM architecture to largely improve data-processing performance and energy efficiency compared to traditional computing architectures. The proposed programming paradigm could also be used in future PIM computing architectures. 

## Figures and Tables

**Table 1:** Configuration detail of the test models.

Architecture	Parameters	
CPU-only	Out-of-Order	
	L1-cache	64 KB
	(64 KB Icache and 64 KB Dcache)	
	L2-cache	1 MB
	block size	64 B
	memory capacity	2 GB
	Clock rate	1 GHz
PIM core	in-order	
	L1-cache	64 KB
	(64 KB Icache and 64 KB Dcache)	
	Clock rate	1 GHz
PIM	CPU-only + one PIM core	
PIM2	CPU-only + two PIM cores	
GPU	NVIDIA GeForce GTX480	
	Fermi GPU architecture	
	15 streaming multiprocessors	
	each containing 32 cores	
	virtual memory page size	4 GB
	Clock rate	700 MHz

**Table 2:** Comparison of cache power consumption between the CPU-only and PIM models running Mpeg2decode programs (mJ).

	Centaur_1.mpg	Cinedemo.m2v
CPU-only	$1.27\times 10^{-2}$	$3.74\times 10^{-2}$
PIM	$2.90\times 10^{-8}$	$6.72\times 10^{-9}$

**Table 3:** Comparison of bus power consumption between the CPU-only and PIM models running Mpeg2decode programs (mJ).

	Centaur_1.mpg	Cinedemo.m2v
CPU-only	$4.33\times 10^{-3}$	$1.80\times 10^{-2}$
PIM	$4.12\times 10^{-8}$	$4.15\times 10^{-8}$

**Table 4:** Memory access latency in the CPU-only model.

	Memory Access Latency	Others
wordcount	24%	76%
histogram	59%	41%
matrix-multiply	69%	31%
string-match	10%	90%

**Table 5:** Comparison of cache power consumption between the CPU-only model and PIM model running MapReduce programs (mJ).

	CPU-Only	PIM
wordcount	0.52	$4.72\times 10^{-5}$
histogram	0.16	$3.87\times 10^{-5}$
matrix-multiply	2.18	$5.82\times 10^{-5}$
string-match	0.13	$4.77\times 10^{-5}$

**Table 6:** Comparison of bus power consumption between the CPU-only model and PIM model running MapReduce programs (mJ).

	CPU-Only	PIM
wordcount	2.93	$6.00\times 10^{-5}$
histogram	0.40	$5.26\times 10^{-5}$
matrix-multiply	0.87	$6.30\times 10^{-5}$
string-match	0.18	$5.92\times 10^{-5}$
